# A Pilot Trial of Molecularly Tailored Therapy for Patients with Metastatic Pancreatic Ductal Adenocarcinoma

**DOI:** 10.1089/pancan.2019.0003

**Published:** 2019-05-02

**Authors:** Anteneh A. Tesfaye, Hongkun Wang, Marion L. Hartley, Aiwu Ruth He, Louis Weiner, Nina Gabelia, Lana Kapanadze, Muhammad Shezad, Jonathan R. Brody, John L. Marshall, Michael J. Pishvaian

**Affiliations:** ^1^ Karmanos Cancer Institute, Wayne State University, Detroit, Michigan.; ^2^ Lombardi Comprehensive Cancer Center, Georgetown University, Washington, District of Columbia.; ^3^ Department of Surgery, Sidney Kimmel Cancer Center, Thomas Jefferson University, Philadelphia, Pennsylvania.

**Keywords:** chemotherapy, metastatic pancreatic cancer, molecular testing

## Abstract

**Purpose:** Despite the wide adoption of tumor molecular profiling, there is a dearth of evidence linking molecular biomarkers for treatment selection to prediction of treatment outcomes in patients with metastatic pancreatic cancer. We initiated a pilot study to test the feasibility of designing a larger phase II trial of molecularly tailored treatment for metastatic pancreatic cancer.

**Methods:** Our study aimed to assess the feasibility of following a treatment algorithm based on the expression of three published predictive markers of response to chemotherapy: ribonucleotide reductase catalytic subunit M1 (for gemcitabine); excision repair cross-complementation group 1 (for platinum agents); and thymidylate synthase (for 5-fluorouracil) in patients with untreated, metastatic pancreatic cancer. Results of the tumor biopsy analysis were used to assign patients to one of seven doublet regimens. Key secondary objectives included response rate (RR), disease control rate (DCR), progression-free survival (PFS), and overall survival (OS).

**Results:** Between December 2012 and March 2015, 30 patients were enrolled into the study. Ten patients failed screening primarily due to inadequate tumor tissue availability. Of the remaining 20 patients, 19 were assigned into 6 different chemotherapy doublets, and achieved an RR of 28%, with a DCR rate of 78%. The median PFS and OS were 5.78 and 8.21 months, respectively.

**Conclusions:** The incorporation of biomarkers into a treatment algorithm is feasible and resulted in a PFS and OS similar to other doublet therapies for patients with metastatic pancreatic cancer. Based on the results from this pilot study, a larger phase II randomized trial of molecularly targeted therapy versus physicians' choice of standard of care has been initiated in the second-line setting (NCT02967770).

## Introduction

Pancreatic cancer is the third leading cause of cancer-related mortality, and it is estimated that 53,670 people were diagnosed with, and 43,090 people died from this disease in 2018. Surgical resection is currently the only potentially curative treatment option, but unfortunately, only 9% and 29% of patients have operable or localized, nonmetastatic disease, respectively. The vast majority of patients are diagnosed with metastatic disease on initial presentation, and these patients have a reported median survival of only 8–11 months.^1–5^

Chemotherapy continues to be the cornerstone of treatment for pancreatic cancer since the approval of gemcitabine in the frontline setting.^2^ Modern day chemotherapy combinations have since improved outcomes over single agent gemcitabine, thereby becoming the new standards of care. In 2011, the combination of 5-fluorouracil (5FU), oxaliplatin, and irinotecan (FOLFIRINOX) was shown to improve median overall survival (OS) to 11.1 months compared with gemcitabine, which demonstrated a median OS of just 6.8 months in patients with a good performance status (ECOG 0-1).^3^ In 2013, the combination of gemcitabine and nab-paclitaxel was also shown to be better than single agent gemcitabine. The median OS of gemcitabine and nab-paclitaxel was 8.5 months, which was statistically superior to the 6.7-month survival seen with gemcitabine.^6^ These chemotherapy regimens in current clinical practice were evaluated empirically in nonbiomarker-enriched patient populations, and there are no accompanying predictive tools to guide their use in patients. Given the short-lived benefit from chemotherapy, it would be a worthwhile endeavor to select patients who are most likely to benefit from a given treatment while sparing treatment-related side effects for those who are less likely to benefit. Although the predictive strength of any single biomarker is debatable,^7^ the utility of a composite of multiple biomarkers in patient selection to match them with best available treatment option(s) has not been widely explored.

Despite the availability of molecular profiling, there is scarcity of high-quality prospective data evaluating the efficacy of linking molecular profiles to specific treatment choices in patients with pancreatic adenocarcinoma. Studies have explored the predictive role of some biomarkers to specific chemotherapeutic agents. One such biomarker is ribonucleotide reductase catalytic subunit M1 (RRM1) expression, which is a potential marker for gemcitabine resistance.^8^ In addition, the expression of excision repair cross-complementation group 1 (ERCC1) and thymidylate synthase (TS) may be markers for platinum resistance^9,10^ and 5FU resistance,^7,11,12^ respectively. We have recently performed a review of the value of these predictive biomarkers across disease types, and our findings suggest that there is value at a minimum to exploring the utilization of these biomarkers in clinical trials for patients with pancreatic and other cancer types.^13^ Examples of the incidence of high or low expression of RRM1, ERCC1, and TS have been previously published. For example, for RRM1, Valsecchi et al. detailed that, of 93 patients assessed, RRM1 expression by immunohistochemistry (IHC) was low in 61 (65%) and high in 32 patients (35%).^14^ Other smaller data sets reveal high expression of RRM1 to be observed in 34 − 50% of patient samples.^15–17^ For ERCC1, Valsecchi et al. detailed that, of 94 patients assessed, ERCC1 expression by IHC was low in 41 (44%) and high in 53 patients (56%).^14^ Fareed et al. and Hwang et al. detailed that high levels of ERCC1 expression were observed in ∼50% of patient samples.^18,19^ Finally, for TS, Hu et al. evaluated pancreatic tumor tissue from 132 resected patients and determined that TS expression was high in 83 of 132 (63%) and low in 49 of 132 patients (37%).^20^ Formentini et al. revealed that TS expression in 130 pancreatic cancer patients was low in 56% and high in 43% of patients.^21^

We designed a pilot study to explore the feasibility of incorporating the use of predictive biomarkers RRM1, ERCC1, and TS to select therapeutic “doublet” chemotherapy for patients with metastatic pancreatic cancer. As per protocol, the stated primary objective was “to determine the estimates of outcomes necessary to plan and conduct subsequent studies with molecularly tailored therapy, for patients with metastatic pancreatic cancer.” More practically stated, the primary objective was essentially to assess the feasibility of testing these markers in newly diagnosed patients, and incorporating the test results into the treatment-decision process. Our key secondary end-points, including disease control rate (DCR), time to progression, and OS, were preliminary assessed to determine the feasibility and efficacy of our “molecularly tailored therapy” selections.

## Patients and Methods

### Patient eligibility

Eligible patients were ≥18 years of age, with cytologically/histologically confirmed metastatic pancreatic adenocarcinoma that was amenable to biopsy to obtain sufficient tissue for molecular profiling. Patients must have not received prior systemic therapy for metastatic disease, have measurable disease by RECIST version 1.1, an Eastern Cooperative Oncology Group (ECOG) performance status of 0–2, and acceptable liver, renal, and hematologic laboratory values. The study protocol (NCT01888978) was approved by the local institutional review board, and all patients provided written informed consent.

### Study design and procedures

**FIG. 1.**
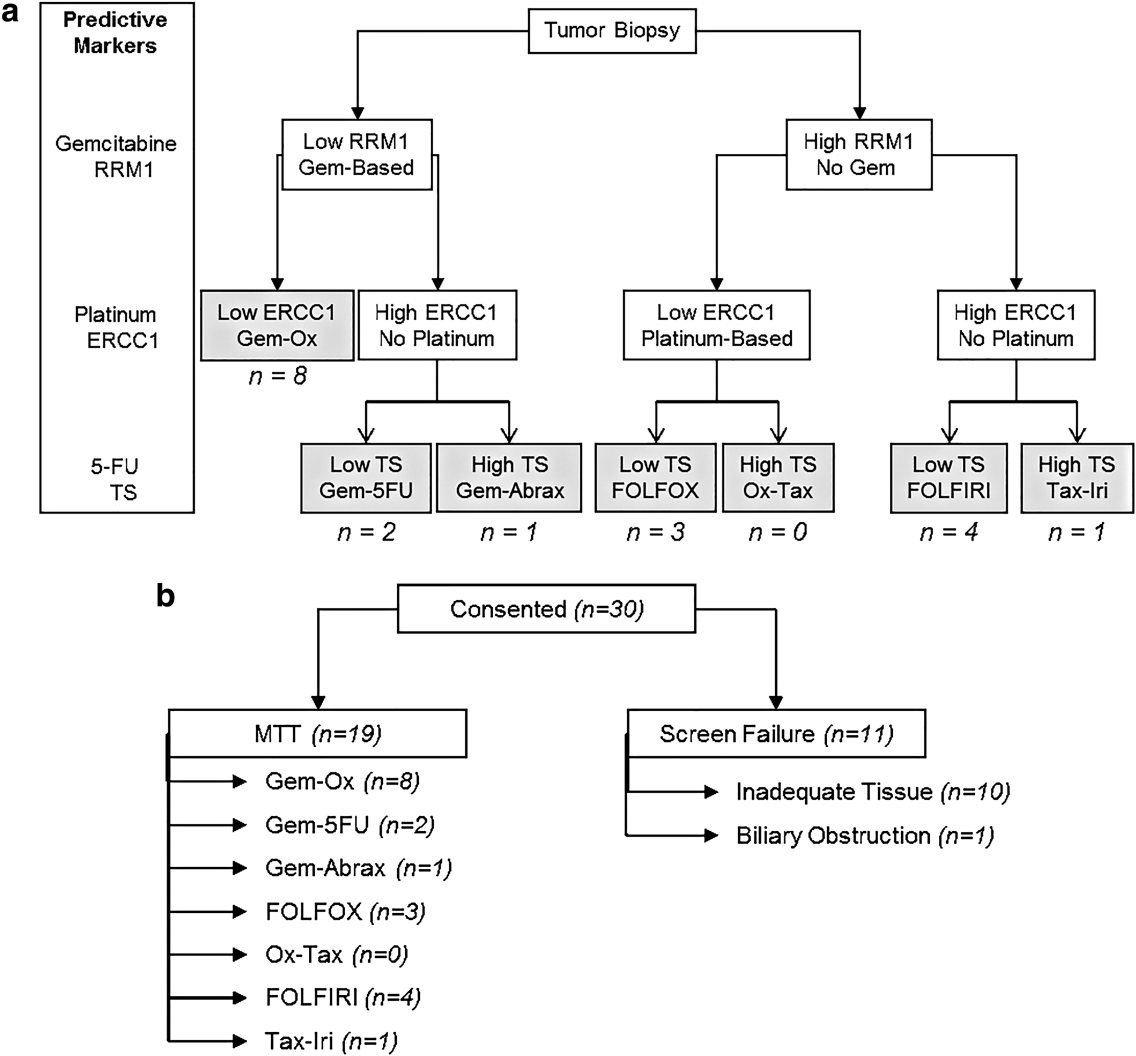
Chemotherapy doublet selection algorithm and patient flow. **(a)** Patients were assigned therapy based on the high versus low values of RRM1, ERCC1, and TS, with cutoffs as determined by Caris. The number of patients (*n*) in each group is also listed. **(b)** The number of patients (*n*) in each group is also listed. 5FU, 5 fluorouracil; Abrax, nab-paclitaxel; ERCC1, excision repair cross-complementation group 1; FOLFOX, 5FU + oxaliplatin; FOLFIRI, 5Fu + irinotecan; Gem, gemcitabine; Iri, irinotecan; Ox, oxaliplatin; RRM1, ribonucleotide reductase catalytic subunit M1; Tax, docetaxel; TS, thymidylate synthase.

This was an open-label pilot study designed to assess the feasibility of following a simple algorithm to treat patients with metastatic pancreatic cancer with a chemotherapy regimen based on three published predictive markers of response/resistance to chemotherapy. As per protocol, the primary objective was “to determine the estimates of outcomes necessary to plan and conduct subsequent studies with molecularly tailored therapy, for patients with metastatic pancreatic cancer.” Biopsies of accessible metastatic lesions were performed by an interventional radiologist under standard biopsy procedures. The specimen was sent to Caris Life Sciences (Caris) for molecular analysis to be checked for expression of, at minimum, RRM1, ERCC1, and TS, although a broader panel was typically assessed. IHC was used to measure the expression of the markers, as per standard operating procedures internal to Caris. For each IHC, the staining intensity and the percent of positive cells was provided by Caris, and the cutoff of “high” versus “low” was determined by Caris, using their internal database. These cutoff values did not change significantly in the course of this study. Patients with inadequate biopsy specimens who were unwilling to undergo a second biopsy were considered screen failures. Patients were assigned a treatment doublet based on expression of RRM1, ERCC1, and TS, using the algorithm depicted below (Fig. 1).

### Treatment details

Patients were treated with one of seven possible chemotherapy doublets, based on their tumor molecular profile (Table 1). Gem-Ox consisted of the combination of 1000 mg/m^2^ gemcitabine on day 1 and 100 mg/m^2^ oxaliplatin on day 2, both given every 14 days,^4,22–24^ and was selected for patients with low RRM1 and ERCC1 expression. Gem-5FU consisted of the combination of 1000 mg/m^2^ gemcitabine and 2000 mg/m^2^ 5FU, given as a 24-h slow infusion, both administered on days 1, 8, and 15 of a 28-day cycle,^1,25–27^ and was selected for patients with low RRM1, high ERCC1, and low TS expression. Gem-Abrax consisted of the combination of 1000 mg/m^2^ gemcitabine and 125 mg/m^2^ nab-paclitaxel, both given on days 1, 8, and 15 of a 28-day cycle,^5^ and was selected for patients with low RRM1, high ERCC1, and high TS expression. FOLFOX consisted of the combination of 85 mg/m^2^ oxaliplatin on day 1, 400 mg/m^2^ 5FU on day 1, 400 mg/m^2^ leucovorin on day 1, and 2400 mg/m^2^ 5FU over 46 h, all administered every 14 days,^28–30^ and was selected for patients with high RRM1, low ERCC1, and low TS expression. Ox-Tax consisted of the combination of 100 mg/m^2^ oxaliplatin on day 1 and 65 mg/m^2^ docetaxel on day 1, both given every 3 weeks with growth factor support,^31^ and was selected for patients with high RRM1, low ERCC1, and high TS expression. 5FU/leucovorin and irinotecan (FOLFIRI) consisted of the combination of 180 mg/m^2^ irinotecan on day 1, 400 mg/m^2^ 5FU on day 1, leucovorin 400 mg/m^2^ on day 1, and 5FU 2400 mg/m^2^ over 46 h, all administered every 14 days,^32–35^ and was selected for patients with high RRM1, high ERCC1, and low TS expression. Finally, Tax-Iri consisted of the combination of 35 mg/m^2^ docetaxel and 50 mg/m^2^ irinotecan, both given weekly for 4 of 6 weeks,^36,37^ and was selected for patients with high RRM1, high ERCC1, and high TS expression. Dose modifications for adverse events were detailed in the protocol for each regimen. Treatment in the assigned subgroup was continued until disease progression or patient intolerance occurred, and all patients were followed until death.

**Table 1.** Selection of Treatment Regimens Based on Tumor Molecular Profile

Regimens	Molecular profile	No. of patients	Refs.
Gem-Ox			
Gemcitabine 1000 mg/m^2^ day 1	Low RRM1	8	^5,22–24^
Oxaliplatin 100 mg/m^2^ day 2	Low ERCC1		
Every 2 weeks			
Gem-5FU			
Gemcitabine 1000 mg/m^2^	Low RRM1	2	^2,25–27^
5-FU 2000 mg/m^2^ 24-h slow infusion	High ERCC1		
Both on days 1, 8, and 15 of a 28-day cycle	Low TS		
Gem-Abrax			
Gemcitabine 1000 mg/m^2^	Low RRM1	1	^28^
Nab-paclitaxel 125 mg/m^2^	High ERCC1		
Both on days 1, 8, and 15 of a 28-day cycle	High TS		
FOLFOX			
Oxaliplatin 85 mg/m^2^ day 1	High RRM1	2	^29–31^
5-Fluorouracil 400 mg/m^2^ day 1	Low ERCC1		
Leucovorin 400 mg/m^2^ day 1	Low TS		
5-Fluorouracil 2400 mg/m^2^ over 46 h			
Every 2 weeks			
Ox-Tax			
Oxaliplatin 100 mg/m^2^ day 1	High RRM1	0	^32^
Docetaxel 65 mg/m^2^ day 1	Low ERCC1		
Mandatory growth factor support	High TS		
Every 3 weeks			
FOLFIRI			
Irinotecan 180 mg/m^2^ day 1	High RRM1	4	^33–36^
Leucovorin 400 mg/m^2^ day 1	High ERCC1		
5-FU 400 mg/m^2^ day 1	Low TS		
5-FU 2400 mg/m^2^ over 46 h			
Every 2 weeks			
Tax-Iri			
Docetaxel 35 mg/m^2^	High RRM1	1	^37,38^
Irinotecan 50 mg/m^2^	High ERCC1		
Weekly for 4 of 6 weeks	High TS		

5FU, 5-fluorouracil; ERCC1, excision repair cross-complementation group 1; RRM1, ribonucleotide reductase catalytic subunit M1; TS, thymidylate synthase.

### Statistical analysis

As a pilot study, no specific statistical hypotheses were tested or were utilized in determining the “estimates of outcomes necessary to plan and conduct subsequent studies.” Rather, descriptive summary statistics were used for patient demographics as well as toxicities (*N*, median, range for continuous variables; and *N*, percent for categorical variables). Kaplan–Meier methodology was used to estimate the progression-free survival (PFS) and OS. Median PFS and OS were presented with their 95% confidence intervals. SAS 9.3 (SAS, Inc., Cary, NC) was used for the analysis.

## Results

Between December 2012 and March 2015, 30 patients with metastatic pancreatic adenocarcinoma were consented, and 19 of these received molecularly tailored therapy as per protocol (Fig. 1a, b). The median age of patients was 63 years (range: 42–76). The majority of the patients were male (63%) and Caucasian (68%). Most patients (63%) had ECOG performance status of 1. The baseline demographics and disease-related characteristics of the patients are summarized in Table 2.

**Table 2.** Patient Demographics

Characteristics	Enrolled patients (*n* = 19)
Age (in years)	
Median (range)	63 (42–76)
Gender, *N* (%)	
Female	7 (36.8)
Male	12 (63.2)
Race, *N* (%)	
Asian	2 (10.5)
Black	4 (21.1)
White	13 (68.4)
Disease stage, *N* (%)	
IV	19 (100.0)
ECOG performance status, *N* (%)	
0	5 (26.3)
1	12 (63.2)
2	2 (10.5)

ECOG, Eastern Cooperative Oncology Group.

Of the 30 patients consented, 20 (67%) had enough tissue available for tumor molecular profiling and consequent treatment assignment. One patient was a screen failure after biopsy due to biliary obstruction. The remaining 19 patients were able to initiate molecularly tailored therapy. The median time from biopsy to treatment initiation was 32.5 days (range: 14–68). The most common tumor molecular profile was low RRM1 and ERCC1 (44.4%), and these patients were treated with a doublet of gemcitabine and oxaliplatin. The second-most commonly seen profile was high RRM1, high ERCC1, and low TS (22.2%), and these patients were treated with a doublet of FOLFIRI.

Patients continued therapy with protocol-defined dose modifications for adverse events until disease progression or intolerance. At the time of submission of this article, all patients had completed follow-up for PFS and OS, with the exception of one patient who moved out of the country and was lost to follow-up after 3 months.

**FIG. 2.**
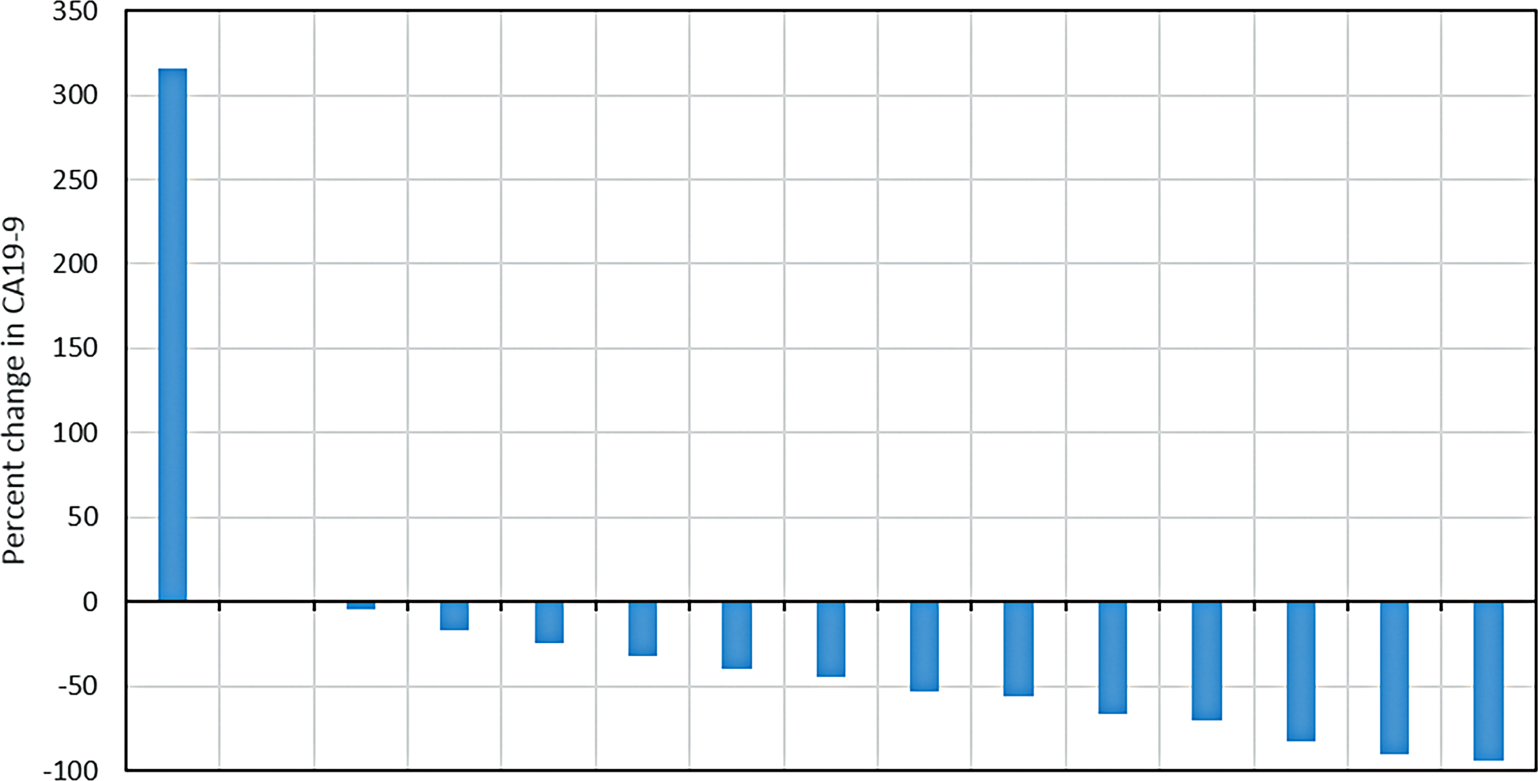
Percent changes in CA19-9 from pretreatment level to the nadir reported on the study.

**FIG. 3.**
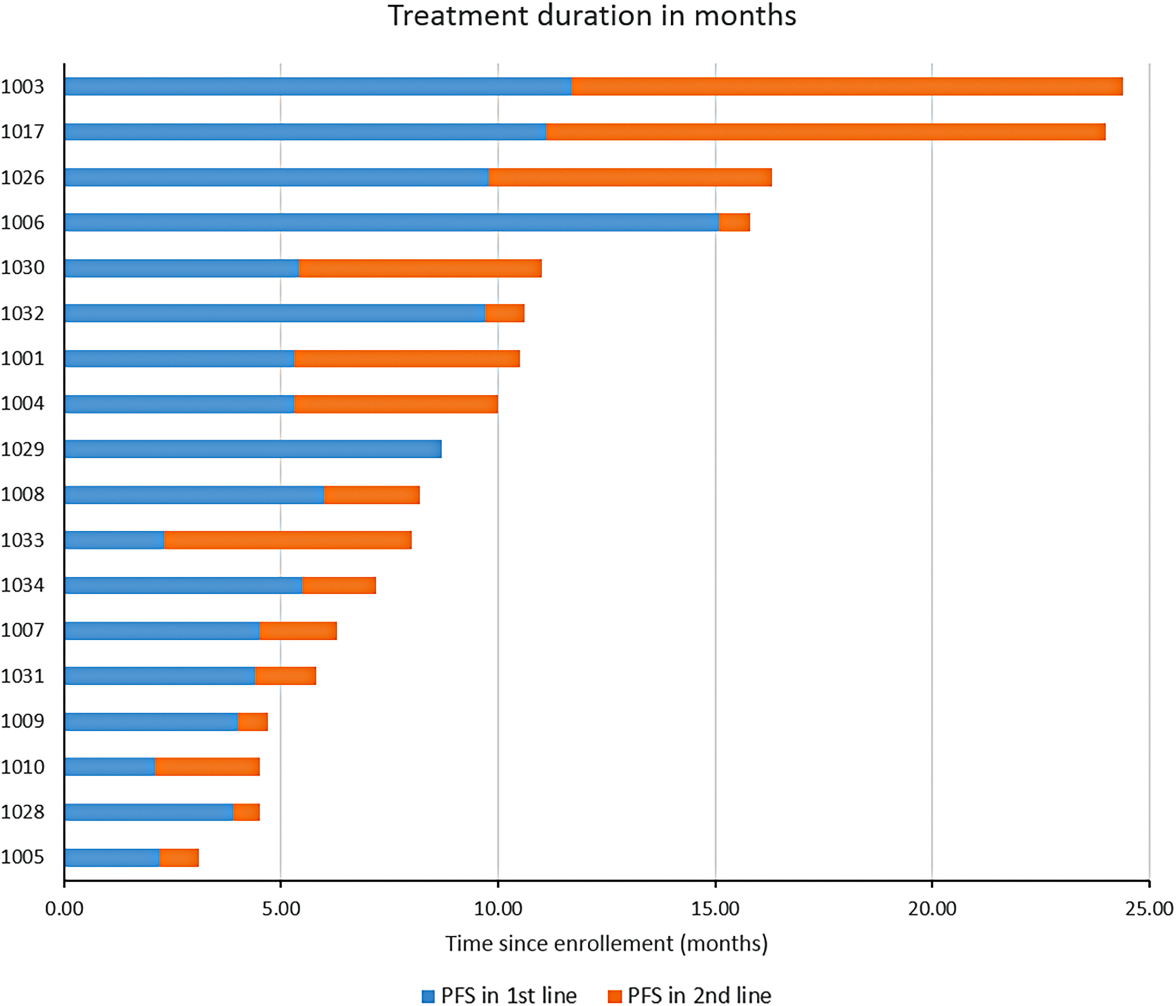
Treatment duration and response by individual patients.

**FIG. 4.**
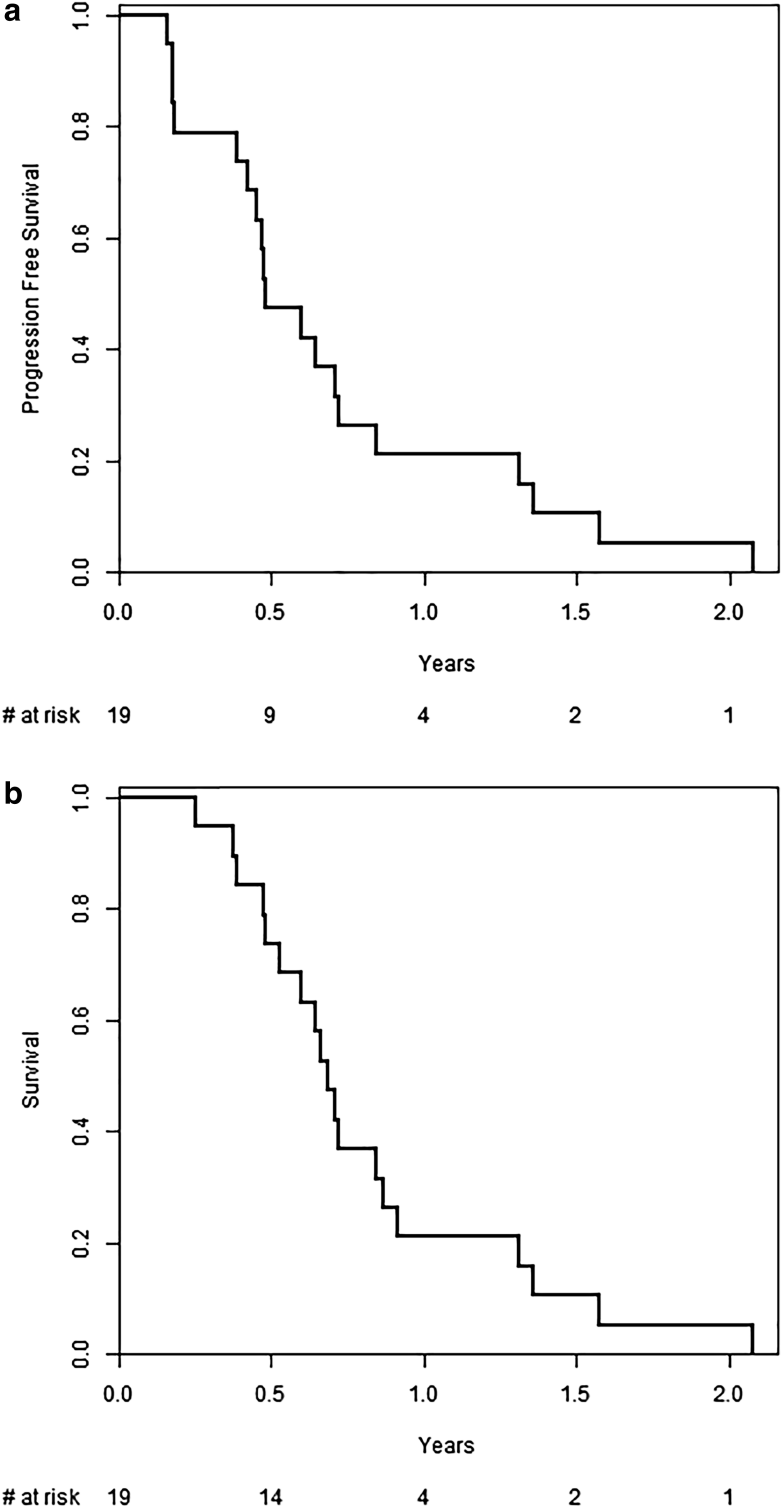
Median PFS and OS. **(a)** Graph showing Kaplan–Meier of PFS. The median PFS (95% CI) was 5.78 months (5.39–15.72). **(b)** Graph showing Kaplan–Meier of OS. The median OS (95% CI) was 8.21 months (7.16–15.72). CI, confidence interval; OS, overall survival; PFS, progression-free survival.

The CA 19-9 best response is shown in Figure 2 (for the 15 patients with baseline elevation in CA 19-9), and 55% of patients had >50% reduction in their tumor marker. The 6-month DCR was 78%, with partial response and stable disease seen in 28% and 50% of patients, respectively. The median PFS was 5.78 months (95% CI 5.39–15.72), while the median OS was 8.21 months (95% CI 7.16–15.72). Treatment durations are depicted in Figure 3. Survival and response details are listed in Table 3, and PFS and OS are depicted in Figure 4a and b.

**Table 3.** Maximum Response: RECIST, CA19-9, and Survival

Response	*N* (%)
RECIST best tumor response	
Disease control rate	14 (78)
Complete response	0 (0)
Partial response	5 (28)
Stable disease	9 (50)
Progressive disease	4 (22)
CA 19-9 reduction (total *n* = 15), *n* (%)	
>20%	15 (83)
>50%	10 (55)
>90%	4 (22)
Survival (months) (95% CI)	
PFS, months	5.78 (5.39–15.72)
OS, months	8.21 (7.16–15.72)
Alive at 6 months, *n* (%)	13 (72)

OS, overall survival; PFS, progression-free survival.

Grade 3 adverse events reported among the patients treated with molecularly tailored regimens included nausea/vomiting (10%), anemia (10%), thrombocytopenia (10%), venous thromboembolism (5%), peripheral neuropathy (5%), and febrile neutropenia (5%). There were no Grade 4 adverse events reported (Table 4 for complete list of adverse events).

**Table 4.** All Cause Adverse Events

Adverse events	All grades (*N*)	Grade 3, *N* (%)
Nonhematologic AE		
Fatigue	13	
Nausea/vomiting	8	2 (10)
Anorexia	4	
Hypoalbuminemia	2	
Weight loss	2	
Constipation	2	
Abdominal distention	1	
Edema	2	
Gastroesophageal reflux	2	
Paresthesia	7	1 (5)
Fever	3	
Alopecia	2	
Dehydration	3	
Hypokalemia	2	
Peripheral sensory neuropathy	2	
Renal calculi/renal colic	1	
Hematuria	1	
Hemorrhage	1	
Cough	1	
Hypoglycemia	1	
Hypotension	1	
Hematologic		
Anemia	4	2 (10)
Platelet count decreased	3	2 (10)
Thromboembolic event	2	1 (5)
Febrile neutropenia	1	1 (5)
Wound infection	1	1 (5)
Catheter-related infection	1	
↑ Alanine aminotransferase	3	1 (5)
↑ Alkaline phosphatase	3	1 (5)
↑ Aspartate aminotransferase	1	1 (5)

There were no Grade 4 adverse events reported.

AE, adverse events.

On disease progression, eight patients (40%) did not receive any further treatment, of whom seven (88%) died within 3 months of failure of the frontline therapy. Twelve patients (60%) underwent second-line therapy at the discretion of the treating physician, of whom four patients (33%) received gemcitabine and nab-paclitaxel, three patients (25%) received FOLFOX/XELOX, two patients (16.7%) received FOLFIRI, two patients (16.7%) pursued further clinical trials (immunotherapy, capecitabine/ruxolitinib), and one patient (8%) received single agent 5FU. Patients who had disease progression after frontline treatment but were able to receive second-line therapy had a median OS with further treatment of 5.4 (range: 0.7–13.4) months.

## Discussion

### Tailoring therapy to the molecular profiles of tumor

Although we have greatly improved our understanding of the molecular etiology of pancreatic adenocarcinoma, this has not translated into clinically meaningful treatment options for patients. In fact, no personalized nor even targeted therapy has resulted in clinically meaningful improvements in patient outcomes. By contrast, novel cytotoxic chemotherapy regimens such as FOLFIRINOX and gemcitabine plus nab-paclitaxel have led to an increased OS, while maintaining a reasonable quality of life.^3,5^ These successes indicate that optimal utilization of cytotoxic agents, including gemcitabine, oxaliplatin, irinotecan, 5FU, and taxanes (nab-paclitaxel, paclitaxel, or docetaxel), can improve patient outcomes. Unfortunately, the success of the various cytotoxic chemotherapy regimens is often diluted when tested in large, unselected patient populations.^1–4,6,38,39^ The inability to translate the significant benefits seen in some patients to the pancreatic cancer population at large is because, in common practice, promising chemotherapy combinations are never administered to select patients who are most likely to respond based on their tumor's molecular profile. However, more data have emerged recently^40^ defining molecular subgroups of patients with pancreatic cancer, who, if appropriately selected, could benefit from currently available therapies. This understanding makes a persuasive argument to enhance patient outcomes by adapting predictive tumor biomarkers that will provide a realistic platform for physicians to select treatment with better precision from an array of available agents and regimens for their patients. We initiated a pilot trial to assess the feasibility of tailoring established and FDA-approved cytotoxic chemotherapies for pancreatic cancer patients through molecular analyses of their tumors.

### Molecular targets and predictors

Recognizing the low probability of a single-tumor biomarker to be predictive of chemotherapy benefit in general, we utilized an algorithm of several published markers with the intent of improving clinical outcomes compared with contemporary, nonbiomarker-enriched treatment strategies.

In this study, patients underwent fresh tumor biopsies and the tumors were assessed for three specific published predictive markers of response to chemotherapy. The first such marker is RRM1, which can predict resistance to gemcitabine.^8^ The overexpression of ERCC1 can lead to resistance to platinum drugs.^9,10^ The expression of TS can predict resistance to 5FU.^7,11,12^ Based on the tumor biomarker profile, the algorithm outlined in Figure 1 was used to assign patients to one of seven possible “doublet” chemotherapy combinations. There are no high-quality prospective data that show a clinically meaningful benefit as a result of using these biomarkers to guide treatment selection. This pilot trial was a feasibility study to plan a larger phase II study of the use of such biomarkers to guide treatment selection for patients with advanced pancreatic cancers.

### Outcomes using molecular predictors to select therapy

Treatment offered to patients based on their molecular profiles was very well tolerated, although it is difficult to compare any specific adverse event to larger studies due to the small number of patients. This trial demonstrated the feasibility of obtaining and analyzing fresh tumor biopsies to guide treatment selection. However, there was a 40% screen failure rate, indicating the constraints of making treatment decisions based on the molecular profile of tumors in previously untreated patients who are likely to have significant disease-related symptoms and may not be able to afford waiting for tumor profiling results before a treatment decision can be made and therapy is initiated.

Moreover, our data suggest a potential benefit to molecularly tailored therapy, with the majority of patients achieving a 6-month DCR of 78%, with partial response and stable disease seen in 28% and 50% of patients, respectively. The majority (55%) of patients had >50% reduction in their tumor marker (CA 19-9). The median PFS of 5.78 months (95% CI 5.39–15.72) and the median OS of 8.21 months (95% CI 7.16–15.72) were similar to other published doublet therapy results.^1,2,4,38^

### Second-line treatment outcomes

A significant portion (40%) of the study population was not able to pursue further treatment for their disease. This underscores the need for effective frontline therapy to potentially benefit these patients, a significant proportion of whom will never be able to pursue treatment in the second-line setting once the frontline regimen fails. These patients may potentially benefit from molecularly tailored treatment strategies, which may help identify regimens that are likely/unlikely to be effective. Contrary to the treatment outcomes in the frontline setting, the effectiveness of chemotherapy beyond the frontline setting sharply declines in patients with metastatic pancreatic carcinoma. Second-line trials historically have shown very low response rates (less than 30%) and OS of 4–6 months.^41–48^ Although the sample was small, our study's findings are in line with those previously described.^41–48^

## Conclusions

The incorporation of biomarkers to guide the selection of chemotherapy is feasible and resulted in a similar PFS and OS compared with other standard therapies for patients with metastatic pancreatic cancer. To investigate the benefit of using this approach, a randomized trial versus standard of care has been developed in the second-line setting, taking into consideration the high rate of screen failures due to inadequate tissue sampling (NCT02967770).

## Abbreviations Used

5FU: 5-fluorouracil
CI: confidence interval
DCR: disease control rate
ECOG: Eastern Cooperative Oncology Group
ERCC1: excision repair cross-complementation group 1
FOLFIRI: 5FU/leucovorin and irinotecan
IHC: immunohistochemistry
OS: overall survival
PFS: progression-free survival
RR: response rate
RRM1: ribonucleotide reductase catalytic subunit M1
TS: thymidylate synthase
